# Associations between anthropometric and body composition indices with subclinical arterial damage in chronic inflammatory diseases

**DOI:** 10.1038/s41366-026-02019-0

**Published:** 2026-02-10

**Authors:** Panagiota Kaloudi, Athanase D. Protogerou, Evaggelia K. Aissopou, Eirini D. Basdeki, Antonios A. Argyris, Elpida Athanasopoulou, Nikolaos Tentolouris, Maria G. Tektonidou, Petros P. Sfikakis, Kalliopi Karatzi

**Affiliations:** 1 Cardiovascular Prevention & Research Unit, Clinic & Laboratory of Pathophysiology, Department of Medicine, National and Kapodistrian University of Athens, Athens, Greece; 2 Laboratory of Dietetics and Quality of Life, Department of Food Science & Human Nutrition, Agricultural University of Athens, Athens, Greece; 3 Hellenic Foundation for Cardiovascular Health and Nutrition, Athens, Greece; 4 First Department of Propaedeutic Internal Medicine, School of Medicine, National and Kapodistrian University of Athens, Laiko General Hospital, Athens, Greece; 5 Joint Academic Rheumatology Program, School of Medicine, National and Kapodistrian University of Athens, Athens, Greece

**Keywords:** Risk factors, Health care

## Abstract

**Background:**

Anthropometric and body composition indices are associated with subclinical arterial damage (SAD) in populations with overweight and obesity, but limited data exist in populations with chronic inflammatory diseases (CID). Herein we tested the hypothesis that the associations of anthropometric and body composition indices with macro- and microcirculation SAD markers differ in patients with CID compared to non-CID subjects.

**Methods:**

A total of 264 patients with CID (23.1% men, mean age ± standard deviation: 51.1 ± 12.9 years) and 491 CID-free individuals with cardiovascular disease risk factors (55.8% men, mean age ± standard deviation: 51.9 ± 11.6 years) were enrolled. Anthropometric measurements, carotid intima-media thickness (IMT), pulse wave velocity (PWV) and retinal vessel calibers were assessed in all participants.

**Results:**

In non-CID individuals, all anthropometric indices were positively associated with IMT; most of them were positively associated with central retinal venular equivalent and inversely associated with central retinal arteriolar equivalent and arteriolar to venular ratio. In patients with CID, only body fat percentage [*B* = 0.002 95% CI (0.000, 0.004)], mid-upper arm circumference [*B* = 0.007 95% CI (0.002, 0.011)] and waist circumference [*B* = 0.001 95% CI (0.000, 0.003)] were positively associated with IMT, whereas none of the indices were associated with retinal microcirculation. No associations with PWV were observed in both groups.

**Conclusions:**

Body weight, body mass index, waist-to-hip ratio and waist-to-height ratio were not associated with SAD markers in patients with CID. This may have an impact on the clinical use of these indices in the presence of CID, which needs to be elucidated by future studies.

## Introduction

Cardiovascular disease (CVD) is a major cause of mortality worldwide [1]. Indices of subclinical arterial damage (SAD) reflect early morphological and functional changes in the arterial wall in macro- and microcirculation and can be applied for the early assessment and timely prevention of CVD risk [2]. Carotid intima-media thickness (IMT) derived by ultrasonography is a widely applied marker of subclinical atheromatosis-hypertrophy that is used for CVD risk reclassification [3]. Carotid-to-femoral pulse wave velocity (cf PWV), as assessed non-invasively using a tonometric method, is the gold standard for aortic stiffness assessment and can effectively reclassify CVD risk [4]. Finally, retinal vessel caliber, such as central retinal arteriolar equivalent (CRAE), central retinal venular equivalent (CRVE) and arteriole-to-venule ratio (AVR) can be measured non-invasively by a computer-assisted method; a narrower CRAE, wider CRVE and a lower AVR predict cardiovascular events [5].

Many indices are used to monitor body composition; most of them are also useful in clinical practice since they are non-invasive and easy to apply. These indices include body mass index (BMI), waist circumference (WC), waist to hip ratio (WHR), waist to height ratio (WHtR) and mid-upper arm circumference (MUAC) that also provide information about fat distribution [6]. Body fat is estimated vaguely using BMI, since for a given BMI, the body fat percentage, defined as the ratio of fat to total body weight, changes and depends on age and other factors affecting body composition [7]. Recent studies have shown that fat accumulation in the upper body is associated with obesity-related comorbidities, whereas fat accumulation in the gluteofemoral area appears to have a protective role on CVD incidence [8]. A systematic review and meta-analysis of 26 cross-sectional and 5 prospective studies that included more than 300,000 adults observed that WHtR is better predictor than WC and BMI for the detection of cardiometabolic risk [9]. In another very recent systematic review and meta-analysis that included 36 cross-sectional and 2 cohort studies with 105,000 to 137,256 participants, WC and WHR were found to be better predictors of CVD incidence compared to BMI [10].

Anthropometric indices such as BMI and WC have been positively associated with IMT in apparently healthy adults [11, 12]. Fewer studies in a population of individuals without established CVD show that BMI is associated with arterial stiffness, whereas others describe that indicators of abdominal fat accumulation, such as WC or WHtR are more strongly associated with PWV than BMI [13–15]. Moreover, several studies have shown that higher BMI levels are associated with unfavorable changes in retinal vascular calibers in individuals without established CVD [16]. The association between other anthropometric and body composition indices and retinal microcirculation has been scarcely examined and only two cross-sectional studies in individuals with CVD risk factors have shown that increased WC and WHR are associated with a larger retinal venular diameter [17, 18].

People with CID present alterations in comparison to healthy individuals regarding their body composition. Specifically, patients with rheumatoid arthritis (RA) have a unique body composition with high levels of fat mass and low muscle tissue, partly attributed to the chronic inflammation [19]. Fewer studies have assessed the body composition in patients with systemic lupus erythematosus (SLE) and it has been shown that they have lower muscle mass than RA patients and patients with juvenile SLE present high fat mass compared with the healthy controls [20–24]. Based on the systematic review and meta-analysis of nine observational studies, patients with systemic sclerosis (SS) have lower muscle tissue and fat mass compared with healthy controls [25]. Data on body composition in patients with spondylarthropathy (SpA) are scarce. In patients with ankylosing spondylitis muscle mass was reduced in two studies but in the study of Plasqui et al. no difference was found between the patients and controls and fat mass was the same in comparison to healthy controls in both of three studies [26–28]. Patients with psoriatic arthritis have altered body composition, which is characterized by high body fat, visceral fat and a loss of muscle mass [29].

Also, patients with CID have a higher burden of CVD compared with the general population, due to traditional CVD risk factors, chronic systemic inflammation and the increased body fat, which contribute to the accelerated SAD [30]. Indices of SAD are widely used in current clinical practice 6 for CVD risk stratification, as the early detection of vascular damage is crucial [31]. However, it remains unclear whether commonly used anthropometric and body composition indices are associated with the acceleration of vascular damage in CID, or whether they are clinically useful in assessing CVD risk in this population. Therefore, the usefulness of these indicators in evaluating vascular risk among patients with CID has not yet been established. Due to a lack of evidence regarding the corresponding associations in populations with CID, the aim of this study was to investigate the possible association between anthropometric and body composition indices and indices of SAD of the macro- and microcirculation in participants with or without CID, free of established CVD.

## Materials and methods

### Study design and study population

The current study is part of a large cross-sectional study conducted at the Cardiovascular Research Laboratory of the First Department of Propaedeutic and Internal Medicine, Laiko General Hospital, Athens, Greece. The study population consisted of 755 individuals, 491 of whom having one or more CVD risk factors (without any CID) and 264 patients with CID, including RA, SLE, SS and SpA. Participants with established CVD (defined as preexisting coronary artery disease, stroke and peripheral arterial disease) were excluded from the analysis. The study groups differed in sex distribution. Specifically, the CID group had a higher representation of women, while the non-CID group had a higher representation of men. All participants visited the laboratory early in the morning, having refrained from food and any vasoactive medication and after 30 min of acclimatization, they underwent anthropometric measurements as well as vascular assessment with non-invasive methods. All participants provided written informed consent according to the World Medical Association Declaration of Helsinki and the protocol was approved by the Laiko Hospital’s institutional review board.

### Definition of CVD risk factors

Hypertension was defined by the use of antihypertensive drugs and/or office blood pressure measurement higher than 139/89 mm Hg average of 3 sequential readings with 1 min interval in the supine position after at least 10 min of rest (Microlife WatchBP Office, Microlife AG, Widnau, Switzerland) [32]. Dyslipidemia was defined as the use of any lipid-modifying drug or low-density lipoprotein cholesterol level >160 mg/dl [33]. Current smoking was defined by the use of at least 1 cigarette per day; ex-smoking was considered as discontinuation for more than 6 months. Required data were retrieved from the medical records of the participants.

### Anthropometric and body composition indices

Participants’ body weight was measured without shoes in the minimum clothing possible, according to the manufacturer’s instructions. Height was measured without shoes, with the participants standing with their shoulders relaxed, their arms hanging freely, and their head in Frankfurt horizontal plane, to the nearest 0.1 cm by using stadiometer SECA 213. BMI was calculated as weight/height^2^ (kg/m^2^). WC was measured to the nearest 0.1 cm with the use of a non-elastic tape. Each subject was measured in a standing position, at the end of a gentle expiration, after placing the measuring tape on a horizontal plane around the trunk, at the level of the umbilicus, midway between the lower ribs and the iliac crest. Hip circumference was measured as the largest circumference between the waist and thighs, both in standing position, taken at the maximum posterior extension of the buttocks. WHR was calculated with the equation: WHR = WC (cm)/hip circumference (cm). MUAC was measured with a non-stretchable measuring tape at a point equidistant between the acromion process of the left scapula and the olecranon process of the left ulna. Body composition was measured using the Tanita TBF-300 (Tanita Corp., Tokyo, Japan) bioelectrical impedance device, by trained research assistants. Participants were instructed to abstain from any food or liquid intake and from any intensive exercise for at least 4 h before measurement. They were also instructed not to wear any metal object during measurement.

### Analysis of retinal vasculature

Both eyes of each participant were photographed with a 45*°* digital non-mydriatic retinal camera (Topcon TRC-NW8, Tokyo, Japan) after 5 min of adaptation in the dark through a validated method [34]. Retinal images were centered on the optic disc, followed by quantitative retinal grading, conducted by a well-trained physician (EKA) blinded to clinical data. For each photograph, the calibers of retinal arterioles and venules passing through a zone between 0.5 and 1.0 disc diameters from the optic disc margin were measured and analyzed using a Static Retinal Vessel Analyzer (SVA-T and Vesselmap 2 software, Visualis, Imedos Systems UG, Jena, Germany) [35]. These measurements were then summarized using formulas described elsewhere [35] to compute the CRAE and the CRVE, representing the average internal caliber of retinal arterioles and venules, respectively. Also, CRAE and CRVE were used to estimate the AVR. The intra-observer reproducibility of retinal vascular measurements was excellent (intraclass correlation coefficients >0.93).

### Vascular assessment

All participants underwent vascular assessment, performed by the same examiner. cf-PWV was assessed using the traditional (not XCEL version) of the SphygmoCor apparatus according to a standard methodology (Sphygmocor; Atcor Medical, Sydney, Australia) and was calculated by the ratio of the estimated pulse transit time and the distance traveled by the pressure wave between the carotid and femoral artery. The traveled distance was measured by a tape and the “subtracted distance” methodology was followed [36]. Pressure waves were first recorded at the carotid artery and then, within a few seconds, at the femoral artery. The time delay between the two waves (transit time) was determined using registration with a simultaneously recorded ECG. Two repeated measurements were performed and their average value was used in the analysis. IMT was measured in a plaque-free site at the far wall of the right and left common carotid artery in end-diastole of the cardiac cycle using semi-automatic software. Two sequential images were obtained and the average measurements were used [37].

### Statistical analysis

Statistical analysis was performed using the SPSS statistical package (IBM, version 21.0; IBM, Armonk, New York, USA). Normality of the variables was tested using the Kolmogorov–Smirnov test. Continuous variables are presented as mean ± standard deviation (SD) and categorical variables as frequencies (%). Multiple linear regression analysis was performed to determine the association of specific anthropometric and body composition indices with retinal vessel calibers, PWV and IMT in participants with CID and in non-CID subjects (with CVD risk factors), also adjusting for potential covariates, including age, sex, smoking, hypertension, dyslipidaemia drugs, hypertension drugs and CRAE or CRVE (depending on the dependent variable) [38]. The potential confounders were selected on the basis of a previous publication [38] and among those variables which had univariate analysis association (*p* < 0.10) with retinal vessel calibers in the present study. The models testing CRAE and CRVE related to anthropometric and body composition markers were additionally adjusted for fellow caliber to provide unbiased and biologically plausible results as suggested by Liew et al. [39]. Results are presented as B coefficients, 95% confidence interval (CI). All tests were two-sided and the level of statistical significance was set at *p* < 0.05. Moreover, we performed additional statistical analysis with comparisons between groups of CID diseases and as there were many differences between CID groups, we repeated the multiple regression analysis adjusting for CID group. Also, we repeated our multiple regression analysis using disease duration as a confounder and we adjusted for mean arterial pressure during the PWV measurement- instead of existence of hypertension. Additionally, stratified analyses were performed by sex, age, or hypertension.

## Results

The study population comprised 491 individuals with CVD risk factors (55.8% men, mean age ± standard deviation: 51.9 ± 11.6 years) and 264 patients with CID (23.1% men, 51.1 ± 12.9 years), including RA, SLE, SS and SpA. The study groups differed in sex distribution, with a higher representation of women in the CID group and a higher representation of men in the non-CID group. More details regarding participants’ characteristics are presented in Table 1.

**Table 1:** General characteristics of the study participants.

	Individuals with CVD risk factors	Chronic inflammatory diseases
*N*	491	264
**Age (years)**	51.9 ± 11.6	51.1 ± 12.9
**Men (%)**	55.8	23.1
**Smoking (%)**	54.4	58.0
**Hypertension (%)**	62.5	29.9
**Dyslipidaemia (%)**	26.5	21.3
**Hypertension drugs (%)**	45.4	40.7
**Dyslipidaemia drugs (%)**	22.6	16.7
**Body weight (kg)**	82.7 ± 18.2	70.1 ± 14.7
**Body mass index (kg/m**^**2**^**)**	28.2 ± 5.1	25.7 ± 4.7
**Body fat (%)**	29.2 ± 8.3	30.9 ± 9.3
**MUAC (cm)**	29.7 ± 3.5	27.5 ± 3.4
**Waist circumference (cm)**	96.5 ± 14.5	87.1 ± 13.1
**Waist to hip ratio**	0.9 ± 0.1	0.9 ± 0.1
**Waist-to-height ratio**	56.5 ± 7.7	52.7 ± 7.5
**IMT (mm)**	0.7 ± 0.1	0.6 ± 0.1
**PWV (m/s)**	8.3 ± 1.6	8.0 ± 1.9
**CRAE μm**	169.9 ± 17.2	178.7 ± 17.5
**CRVE μm**	207.8 ± 18.7	211.3 ± 18.0
**AVR**	0.8 ± 0.1	0.8 ± 0.1
**Specific disease**		
Rheumatoid arthritis	-	26.5
Systemic lupus erythematosus	-	24.6
Systemic sclerosis	-	34.1
Spondylarthropathy	-	14.8

Values are presented as mean ± standard deviation or percentage (%)

*CVD* cardiovascular disease, *CRAE* central retinal arteriolar equivalent, *CRVE* central retinal venular equivalent, *AVR* arteriolar to venular ratio, *MUAC* mid upper arm circumference, *PWV* pulse wave velocity, *ΙΜΤ* intima-media thickness.

Table 2 presents the associations between anthropometric and body composition indices and retinal vessel calibers. After adjustment for age, sex, smoking, presence of hypertension, dyslipidaemia, anti-hypertensive drugs and for the fellow caliber (CRAE or CRVE depending on the dependent variable), the following were observed: (i) in individuals with CVD risk factors, all anthropometric and body composition indices were positively associated with CRVE and inversely associated with CRAE (except for MUAC and WHR) and AVR (except for WHR), (ii) no association at all were found in the group of CID patients. Stratified analyses by sex, or age, or hypertension were conducted and after adjusting for disease duration and after using mean arterial pressure during the PWV measurement instead of existence of hypertension as a confounder, no alterations were observed in our findings following these additional analyses.

**Table 2:** Associations between anthropometric and body composition indices and indices of retinal microcirculation.

Individuals with CVD risk factors										Chronic inflammatory diseases								
Anthropometric and body composition indices	CRAE			CRVE			AVR			CRAE			CRVE			AVR		
	*B* (95% CI)	*p* value	Adjusted R2	*B* (95% CI)	*p* value	Adjusted *R*^2^	*B* (95% CI)	*p* value	Adjusted *R*^2^	*B* (95% CI)	*p* value	Adjusted R2	*B* (95% CI)	*p* value	Adjusted *R*^2^	*B* (95% CI)	*p* value	Adjusted *R*^2^
**Body weight (kg)**	**−0.085**	**0.019**	0.504	**0.101**	**0.014**	0.461	**0.000**	**0.011**	0.075	**−**0.028	0.649	0.456	0.064	0.347	0.374	0.000	0.380	0.100
	**(−0.155, −0.014)**			**(0.021, 0.182)**			**(−0.001, 0.000)**			(**−**0.149, 0.093)			(**−**0.070, 0.197)			(**−**0.001, 0.000)		
**Body mass index**	**−0.260**	**0.022**	0.505	**0.256**	**0.047**	0.459	**−0.001**	**0.019**	0.075	**−**0.107	0.548	0.456	0.250	0.202	0.376	**−**0.001	0.251	0.098
**(kg/m**^**2**^**)**	**(−0.482, −0.038)**			**(0.003, 0.509)**			**(−0.003, 0.000)**			(**−**0.455, 0.242)			(**−**0.135,0.635)			(**−**0.003, 0.001)		
**Body fat (%)**	**−0.274**	**0.001**	0.521	**0.312**	**0.002**	0.476	**−0.001**	**0.001**	0.090	−0.026	0.795	0.462	0.067	0.552	0.373	0.000	0.617	0.104
	**(−0.442, −0.106)**			**(0.119, 0.504)**			**(−0.002, −0.001)**			(**−**0.224, 0.172)			(**−**0.155, 0.289)			(**−**0.001, 0.001)		
**MUAC (cm)**	−0.303	0.074	0.498	**0.445**	**0.022**	0.456	**−0.002**	**0.023**	0.071	**−**0.090	0.715	0.454	0.281	0.298	0.373	**−**0.001	0.395	0.100
	(−0.634, 0.029)			**(0.065, 0.824)**			**(−0.004, 0.000)**			(**−**0.572, 0.393)			(**−**0.250, 0.813)			(**−**0.004, 0.001)		
**Waist**	**−0.097**	**0.038**	0.497	**0.139**	**0.010**	0.455	**−0.001**	**0.014**	0.072	**−**0.035	0.631	0.455	0.064	0.422	0.373	0.000	0.398	0.096
**circumference (cm)**	**(−0.189, −0.005)**			**(0.034, 0.244)**			**(−0.001, 0.000)**			(**−**0.176, 0.107)			(**−**0.093, 0.221)			(**−**0.001, 0.000)		
**Waist to hip ratio**	−12.953	0.147	0.495	18.603	0.068	0.452	**−**0.081	0.080	0.065	**−**8.737	0.450	0.455	9.970	0.437	0.373	**−**0.054	0.367	0.096
	(−30.486, 4.580)			(−1.380, 38.585)			(**−**0.172, 0.010)			(**−**31.454, 13.981)			(**−**15.238,			(**−**0.171, 0.063)		
													35.178)					
**Waist to height**	**−0.162**	**0.045**	0.497	**0.206**	**0.026**	0.453	**−0.001**	**0.023**	0.070	**−**0.080	0.495	0.455	0.135	0.296	0.374	**−**0.001	0.279	0.098
**ratio**	**(−0.320, −0.003)**			**(0.024, 0.387)**			**(−0.002, 0.000)**			(**−**0.310, 0.150)			(**−**0.119, 0.390)			(**−**0.002, 0.001)		

Adjusted for age, sex, smoking status, hypertension, dyslipidaemia drugs, hypertension drugs and CRAE or CRVE (depending on the dependent variable).

*CVD* cardiovascular disease, *CRAE* central retinal arteriolar equivalent, *CRVE* central retinal venular equivalent, *AVR* arteriolar to venular ratio, MUAC mid upper arm circumference.

The bold values indicate statistically significant differences (*p* < 0.05) and the B coefficients are also shown in boldfor clarity.

Table 3 presents the associations between anthropometric and body composition indices and indices of early SAD of the macrocirculation. There was a positive association of all anthropometric and body composition markers and IMT after adjustment for age, sex, smoking, presence of hypertension and drugs for dyslipidaemia and hypertension in non-CID subjects. In patients with CID, only body fat percentage [*B* = 0.002 95% CI (0.000, 0.004)], MUAC [*B* = 0.007 95% CI (0.002, 0.011)] and WC [*B* = 0.001 95% CI (0.000, 0.003)] were positively associated with IMT. No association was found between any anthropometric and body composition marker and PWV. Stratified analyses by sex, or age, or hypertension were conducted and after adjusting for disease duration and after using mean arterial pressure during the PWV measurement instead of existence of hypertension as a confounder, no significant changes were observed in our findings.

**Table 3:** Associations between anthropometric and body composition indices and indices of microcirculation.

Anthropometric and body composition indices	Individuals with CVD risk factors						Chronic inflammatory diseases					
	PWV			IMT			PWV			IMT		
	*B* (95% CI)	*p* value	Adjusted *R*^2^	B (95% CI)	*p* value	Adjusted R2	*B* (95% CI)	*p* value	Adjusted R2	*B* (95% CI)	*p* value	Adjusted *R*^2^
**Body weight (kg)**	0.005	0.360	0.327	**0.001**	**0.000**	0.305	0.004	0.569	0.422	0.001	0.064	0.441
	(−0.005, 0.014)			**(0.001, 0.002)**			(−0.010, 0.018)			(0.000, 0.002)		
**Body mass index**	−0.006	0.728	0.326	**0.004**	**0.000**	0.304	−0.001	0.968	0.424	0.003	0.139	0.435
**(kg/m**^**2**^**)**	(−0.039, 0.027)			**(0.002, 0.006)**			(−0.042, 0.041)			(−0.001, 0.006)		
**Body fat (%)**	0.004	0.696	0.317	**0.002**	**0.010**	0.286	0.010	0.374	0.419	**0.002**	**0.025**	0.446
	(−0.017, 0.025)			**(0.001, 0.004)**			(−0.012, 0.031)			**(0.000, 0.004)**		
**MUAC (cm)**	−0.013	0.568	0.325	**0.005**	**0.006**	0.297	−0.009	0.760	0.419	**0.007**	**0.004**	0.457
	(−0.059, 0.032)			**(0.001, 0.008)**			(−0.065, 0.048)			**(0.002, 0.011)**		
**Waist circumference**	0.001	0.930	0.323	**0.002**	**0.000**	0.310	0.006	0.506	0.420	**0.001**	**0.049**	0.441
**(cm)**	(−0.011, 0.013)			**(0.001, 0.003)**			(−0.011, 0.022)			**(0.000, 0.003)**		
**Waist to hip ratio**	−0.228	0.825	0.321	**0.338**	**0.000**	0.308	1.799	0.178	0.424	0.115	0.303	0.431
	(−2.248, 1.793)			**(0.164, 0.513)**			(−0.822, 4.420)			(−0.105, 0.334)		
**Waist-to-height ratio**	−0.008	0.438	0.324	**0.003**	**0.000**	0.307	0.003	0.821	0.420	0.002	0.107	0.436
	(−0.029, 0.013)			**(0.001, 0.005)**			(−0.024, 0.030)			(0.000, 0.004)		

Adjusted for age, sex, smoking status, hypertension, dyslipidaemia drugs and hypertension drugs.

CVD cardiovascular disease, MUAC mid upper arm circumference, PWV pulse wave velocity, ΙΜΤ intima-media thickness.

The bold values indicate statistically significant differences (*p* < 0.05) and the B coefficients are also shown in boldfor clarity.

Supplementary Table 1 presents the differences between CID groups among general characteristics, anthropometric and body composition indices and indices of early SAD of the macro- and microcirculation. Due to the significant differences between the tested groups, multiple regression analyses were repeated using CID group as an additional confounder, yet no change was observed in the results.

## Discussion

To the best of our knowledge, this is the first cross-sectional study that investigated the associations between a variety of anthropometric and body composition indices (body weight, BMI, WC, WHR, WHtR, MUAC, body fat percentage) and indices of early arterial damage (IMT, PWV, CRAE, CRVE, AVR) in a large population of patients with CID as well as in a large population of CID-free individuals with CVD risk factors. Our main findings are that: (i) at the level of the retinal microcirculation, these associations are completely abolished only in the CID patients, and (ii) at the level of the macrocirculation, these associations are present in patients with CID but only for some of the studied anthropometric and body composition indices.

In non-CID participants, our findings are in accordance with the results of the available literature in both the macro- and microcirculation [11, 12, 16–18]. These associations highlight the importance of anthropometric and body composition indices in clinical practice as predictors of SAD in individuals with CVD risk factors. Furthermore, our findings indicate no significant association between anthropometric and body composition indices and PWV. Apart from confounding factors, including age, blood pressure, and physical activity, the most important factor is the carotid-femoral distance measurement, which must be incorporated into the calculation of PWV. In our study, this measurement was carefully standardized to minimize measurement bias; however, minor variability may still occur, particularly in individuals with increased adiposity, as obesity can lead to overestimation of this distance, potentially resulting in miscalculation of PWV [40, 41]. Based on the literature, this association remains unclear since the findings are inconsistent [42–46].

A possible explanation for the diverging findings in patients with CID is probably their modified body composition compared to their healthy counterparts. Specifically, according to the study participants’ characteristics, patients with CID have much lower body weight, BMI and MUAC and slightly higher body fat percentage compared to patients without CID. These findings align with existing literature, including cohort studies, case-control studies, cross-sectional studies, which demonstrate that patients with CID have a different body composition characterized by increased fat mass and reduced muscle tissue compared to patients without CID [19–29, 47]. Notably, MUAC which is a useful tool as an indicator of malnutrition, reflecting muscle protein stores and the abnormal distribution of subcutaneous fat in the upper body [48] and was much lower in CID patients, indicating a loss of muscle mass which is in line with previous studies in CID [49, 50]. In addition, based on our data, patients without CID have much higher mean WC, which should be taken into consideration as it is well recognized as an indicator of accumulated body fat in the abdomen and can predict CVD and SAD risk [10], but it appears to be insufficient for evaluating fat accumulation in CID patients. These alterations are mainly attributed to the presence of chronic systemic inflammation (RA, SLE, SS, and SpA) and are related to disease factors (disease duration, disease activity, glucocorticoid therapy) and several other factors, such as co-morbidities, physical inactivity and malnutrition [51, 52]. Hence, the release of pro-inflammatory cytokines in combination with all the above-mentioned factors possibly negated the association of anthropometric and body composition indices with markers of early arterial damage in patients with CID.

It is noteworthy that patients with CID have increased risk of CVD in comparison with the general population, potentially due to the chronic systemic inflammation, the disease-related treatments as well as the increased prevalence of CVD risk factors (including the associated high fat mass) [53–55]. In particular, disease duration is an important predictor of cardiovascular risk in patients with RA, with several studies demonstrating that mortality increases with longer disease duration. This association is well established in RA and several studies suggest that it can be used to other CID [56]. Accordingly, disease duration was included in our analysis and no significant changes were observed in the results, indicating that our findings are independent of disease duration. Pharmacological treatments vary between CID groups, reflecting differences in both the underlying conditions and the therapeutic needs of patients. Different pharmacological approaches have been described in the literature, partly depending on individual patient and disease-specific characteristics [57]. In our analysis, we adjusted for the medication use as a potential confounder and no significant changes were observed in the results.

In the last decade, the research interest has been focused on investigating body composition markers that assess early vascular damage. According to a recent cross-sectional study that included 197 women (100 RA patients and 97 age-matched controls), fat accumulation is not associated with endothelial dysfunction in RA patients, but it is the loss of muscle mass that is probably associated with endothelial dysfunction in this population [58]. Also, few recent cross-sectional studies found that epicardial fat thickness can be used as a predictor of SAD in patients with RA, SLE, SS and SpA [59–65]. Regarding other novel anthropometric indices based on the statement by the European Society of Cardiology, perivascular and pericardial adipose tissue may be used for the CVD risk assessment but their clinical practice is still uncertain [66]. Therefore, it is important to highlight the need for assessing risk of CVD not merely through classic indices (i.e., body fat, percentage of body fat) but also with the estimation of fat-free mass, body composition changes and novel ones (such as epicardial fat) in populations with CID.

The present study has both strengths and limitations. Τhe main strength is the novelty of the findings. In addition, patients’ medical histories were obtained from verified medical records, and anthropometric as well as body composition indices were measured directly by the researchers rather than being self-reported. However, the cross-sectional design of the study does not allow for the establishment of causal relationships. Another limitation is the heterogeneity of the study population, which included individuals with different CID. Moreover, the study groups differed in sex distribution, with a higher representation of women in the CID group and a higher representation of men in the non-CID group, which can be partly explained by the higher prevalence of CID in women.

## Conclusion

In conclusion, our findings suggest that anthropometric and body composition indices do not provide the same information in CID as in non-CID individuals regarding the presence of retinal microcirculation and carotid artery disease, probably due to the presence of chronic systematic inflammation or other CID-specific factors. This may have an impact on the clinical use of these indices, especially regarding the assessment of vascular damage and its use in CVD risk stratification in these populations. Future studies are needed to confirm the results of the current study, clarify the possible deviations and identify body composition indices that can be used to monitor vascular health in patients with CID.

## Supplementary information

Supplementary Table 1

## Data Availability

The datasets generated during and/or analyzed during the current study are available from the corresponding author on reasonable request.
