# Supplementary material for: Associations between anthropometric and body composition indices with subclinical arterial damage in chronic inflammatory diseases
Source: Int J Obes (Lond). 2026 Feb 10;50(4):887–94. doi: 10.1038/s41366-026-02019-0 (PMC13056531; doi:10.1038/s41366-026-02019-0)
Supplement: Supplementary file 1 — Supplementary Table 1 [file 41366_2026_2019_MOESM1_ESM.pdf]

Supplementary Table 1: Comparisons between groups of CID diseases among general characteristics, anthropometric and body composition indices and indices of macro- and microcirculation

N = 264	Rheumatoid arthritis N=70	Systemic lupus erythematosus N=65	Systemic sclerosis N=90	Spondyloarthropathy N=39	p value
**Age (years)**	56±10.7	43.6±11.5	53.2±13.5	50. 1±12.0	**0.000**
**Men (%)**	20.0	9.2	11.1	79.5	**0.000**
**Hypertension drugs (%)**	27.1	37.5	60.0	25.6	0.000
**Dyslipidaemia drugs (%)**	24.3	7.8	16.7	17.9	0.087
**SBP (mm Hg)**	124.2±18.5	121.8±17.3	119.6±16.9	124.9±15.1	0.269
**DBP (mm Hg)**	76.5±13.0	73.8±10.3	69.9±7.9	78.0±10.2	**0.000**
**Body weight (kg)**	71.1±14.9	67.8±14.9	65.9±11.9	82.5±13.5	**0.000**
**Body mass index (kg/m2)**	26.7±4.8	25.4±5.4	24.7±4.2	27.1±3.4	**0.010**
**Body fat (%)**	33.1±8.3	30.8±10.1	31.0±8.8	27.3±9. 6	**0.022**
**MUAC (cm)**	27.7±3.4	27.1±3.9	26.9±3.1	29.0±2.7	**0.009**
**Waist circumference (cm)**	89.5±11.9	83.3±13.4	83.0±10.5	98.5±12.9	**0.000**
**Waist to hip ratio**	0.9±0.1	0.8±0.1	0.8±0.1	1.0±0.1	**0.000**
**Waist to height ratio**	54.5±6.8	51.0±8.7	50.9±6.5	56.6±6.8	**0.000**
**IMT (mm)**	0.7±0.1	0.6±0.1	-*	0.7±0.2	**0.000**
**PWV (m/sec)**	8.2±1.5	7.4±1.8	8.2±2.1	8.2±1.7	0.055
**CRAE (μm)**	172.9±18.5	180.4±16.1	184.4±16.5	172.9±15 .5	**0.000**
**CRVE (μm)**	209.1±18.9	213.4±16.7	212.0±17.6	210.0±19.3	0.513
**AVR**	0.8±0.1	0.9±0.1	0.9±0.1	0.8±0.1	**0.000**

Values are presented as mean ± standard deviation and p-value or percentage (%)

SBP: systolic blood pressure, DBP: diastolic blood pressure, CVD: cardiovascular disease, CRAE: central retinal arteriolar equivalent, CRVE: central retinal venular equivalent, AVR: arteriolar to venular ratio,

MUAC: mid upper arm circumference, PWV: pulse wave velocity, IMT: intima-media thickness

*not available at this population
